# Diplectanids from *Mycteroperca* spp. (Epinephelidae) in the Mediterranean Sea: Redescriptions of six species from material collected off Tunisia and Libya, proposal for the '*Pseudorhabdosynochus riouxi* group’, and a taxonomic key

**DOI:** 10.1371/journal.pone.0171392

**Published:** 2017-02-02

**Authors:** Amira Chaabane, Lassad Neifar, Jean-Lou Justine

**Affiliations:** 1 Laboratoire de Biodiversité et Écosystèmes Aquatiques, Faculté des Sciences de Sfax, Université de Sfax, Sfax, Tunisie; 2 ISYEB, Institut Systématique, Évolution, Biodiversité, UMR7205 (CNRS, EPHE, MNHN, UPMC), Muséum National d’Histoire Naturelle, Sorbonne Universités, Paris, France; Institut national de la santé et de la recherche médicale - Institut Cochin, FRANCE

## Abstract

Diplectanid monogeneans are gill parasites that can infect fish in huge numbers and thus become harmful, especially in maricultured fish. It is therefore useful to have taxonomic tools, such as keys, to identify species. The following diplectanid species from groupers of the Mediterranean Sea were studied: five species of *Pseudorhabdosynochus* Yamaguti, 1958, including *P*. *riouxi* (Oliver, 1986) Kritsky & Beverley-Burton, 1986 from the dusky grouper *Mycteroperca marginata*, *P*. *enitsuji* Neifar & Euzet, 2007, *P*. *bouaini* Neifar & Euzet, 2007, *P*. *dolicocolpos* Neifar & Euzet, 2007 and *P*. *sinediscus* Neifar & Euzet, 2007 from the goldblotch grouper *M*. *costae*, and *Echinoplectanum echinophallus* (Euzet & Oliver, 1965) Justine & Euzet, 2006 from the dusky grouper. New material was obtained from fish collected from off Tunisia and Libya and compared to the type-material and voucher specimens in museum collections. Identifications of fish were confirmed by barcoding of cytochrome c oxidase subunit I (COI) sequences. The sclerotized vagina was considered the most important structure for systematics. The three species *P*. *riouxi*, *P*. *bouaini*, and *P*. *enitsuji* share a common general structure of the sclerotized vagina with a conspicuous spherical secondary chamber. We thus propose the ‘*Pseudorhabdosynochus riouxi* group’ to accommodate them. *Pseudorhabdosynochus dolicocolpos* has an elongate vaginal structure that is completely different from all its congeneric species reported from the Mediterranean Sea, and *Pseudorhabdosynochus sinediscus* has a sclerotized vagina in which the secondary chamber is not visible, and a haptor without squamodiscs. A taxonomic key to diplectanid species on *Mycteroperca* spp. in the Mediterranean Sea is proposed; it includes ten species of *Pseudorhabdosynochus* and one species of *Echinoplectanum*.

## Introduction

Diplectanid monogeneans are ectoparasites on the gills of fish, generally smaller than 1 mm, but often present in high numbers, even on wild fish. Some diplectanid species can pullulate on fish in confinement and thus become a major health problem for fish in aquaculture [1–5]. On groupers especially, several diplectanid species have been reported as a concern for mariculture [6–11]. It is therefore important to provide taxonomic identification keys to parasite species, which are currently lacking for diplectanids from groupers in the Mediterranean Sea.

Diplectanids from groupers are currently classified into four genera [12]: mostly *Pseudorhabdosynochus* Yamaguti, 1958, with about eighty described species and, with a much smaller number of species, *Laticola* Yang et al., 2006 [13–15], *Echinoplectanum* Justine & Euzet, 2006 [16] and *Diplectanum* Diesing, 1858 [14, 17–20]. Of these four genera, only *Pseudorhabdosynochus* and *Echinoplectanum* are currently reported in groupers in the Mediterranean Sea.

Species of *Pseudorhabdosynochus* Yamaguti, 1958 occur in the tropical and subtropical regions [12, 21–26]. The highest levels of diversity have been recorded especially in shallow-water coral-reef fish [12, 14], while deep-sea groupers show lower parasite diversity [15, 21, 25, 27–29]. The comparative morphology of the sclerotized vaginal parts has proven useful for identifying species within *Pseudorhabdosynochus*, although other sclerotized parts, such as the male copulatory organ, the squamodiscs and the haptoral parts, are also important characters.

This study was undertaken to further examine the sclerotized vaginae of some species of *Pseudorhabdosynochus* previously described from two grouper hosts, the dusky grouper *Mycteroperca marginata* (Lowe) and the goldblotch grouper *M*. *costae* (Steindachner), in the Mediterranean Sea. These are *P*. *riouxi* (Oliver, 1986) Kritsky & Beverley-Burton, 1986 from *M*. *marginata*, and *P*. *enitsuji* Neifar & Euzet, 2007, *P*. *bouaini* Neifar & Euzet, 2007, *P*. *dolicocolpos* Neifar & Euzet, 2007, and *P*. *sinediscus* Neifar & Euzet, 2007 from *M*. *costae*. With the exception of *P*. *riouxi*, none of these species has been reported since their description [24, 30].

On the basis of type-material and specimens newly collected from off Tunisia and Libya (North Africa), we found that *P*. *riouxi*, *P*. *bouaini*, and *P*. *enitsuji* are morphologically close. The three species exhibit a sclerotized vagina with a conspicuous spherical secondary chamber. Hence, the ‘*P*. *riouxi* group’ is proposed here to accommodate them. In contrast, *P*. *dolicocolpos* and *P*. *sinediscus* are separated from all Mediterranean congeners by their unique vaginal structure. A key for diplectanid species of *Mycteroperca* spp. in the Mediterranean Sea is provided.

For fish identifications by barcoding, the Cytochrome c Oxidase subunit I (COI) sequences from our sampled specimens were compared with other COI sequences from different geographical regions published in GenBank; sequences and detailed comparisons were previously published [22, 31, 32].

## Materials and methods

### Fish sampling and identification

Fish were purchased at the fish markets in Sfax, Tunisia and Tripoli, Libya. These were previously caught by fishermen in the nearby coastal waters of the Mediterranean Sea. In all cases, the fish were dead when available for parasitological studies. Fish were identified morphologically according to keys [33, 34], and these identifications were challenged by the COI sequences of individual fish (Table 1). Fish nomenclature follows [35] and [36].

**Table 1:** Fish examined and monogeneans collected.

*Mycteroperca* host species	Locality	Date	Fish COI, GenBank	Reference for fish identification by COI	Diplectanid monogeneans collected
*M*. *marginata*	Tunisia	25/09/2014	KX255749	[22]	*P*. *beverleyburtonae* *P*. *riouxi* *E*. *echinophallus*
*M*. *marginata*	Tunisia	25/07/2015	KU739518	[31]	*P*. *beverleyburtonae* *P*. *riouxi* *E*. *echinophallus*
*M*. *marginata*	Tunisia	22/10/2015	KU739521	[31]	*P*. *beverleyburtonae* *P*. *riouxi* *E*. *echinophallus*
*M*. *costae*	Tunisia	13/06/2014	KX255750	[22]	*P*. *sosia* *P*. *bouaini* *P*. *enitsuji* *P*. *dolicocolpos* *P*. *sinediscus*
*M*. *costae*	Tunisia	13/06/2014	KX255751	[22]	*P*. *sosia* *P*. *bouaini* *P*. *enitsuji* *P*. *dolicocolpos*
*M*. *costae*	Tunisia	15/04/2014	KT805240	[32]	*P*. *sosia* *P*. *bouaini* *P*. *enitsuji* *P*. *dolicocolpos* *P*. *sinediscus*
*M*. *costae*	Tunisia	17/09/2015	KX255747	[22]	*P*. *sosia* *P*. *bouaini* *P*. *enitsuji* *P*. *dolicocolpos* *P*. *sinediscus*
*M*. *costae*	Libya	2013	-		*P*. *sosia* *P*. *bouaini* *P*. *enitsuji* *P*. *dolicocolpos*

### Monogenean morphology

**Fig 1:**
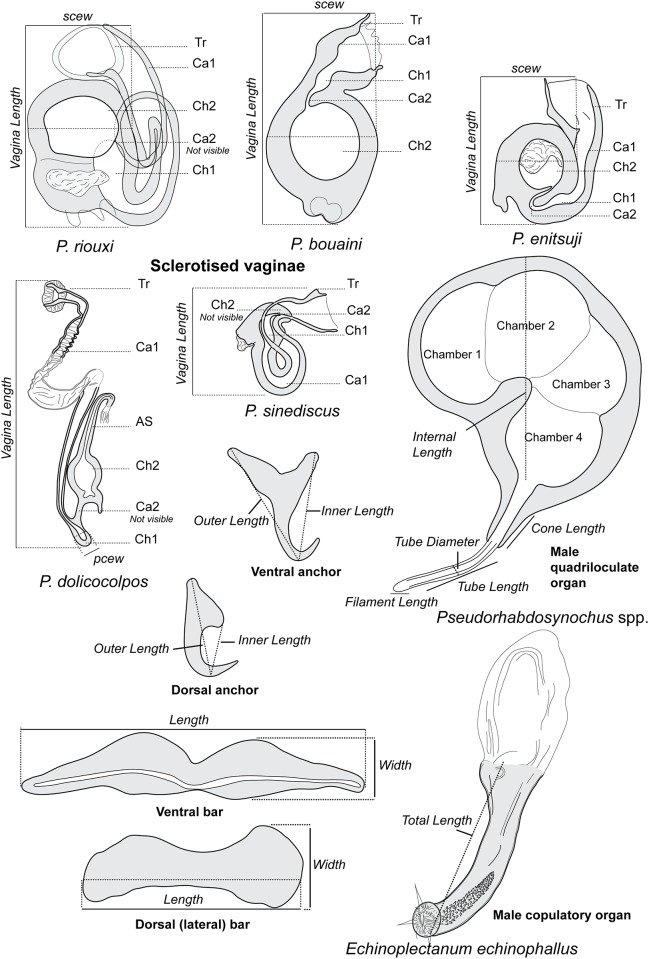
Nomenclature of parts and methods of measurements of sclerotized parts. The diagrams indicate the nomenclature used in this paper and the method of measurements for all sclerotized parts, including male and female organs and haptoral parts for *Pseudorhabdosynochus* spp., and male copulatory organ for *Echinoplectanum echinophallus*. Abbreviations: AS, accessory structure; Ca1, primary canal; Ca2, secondary canal; Ch1, primary chamber; Ch2, secondary chamber; Tr, trumpet. Measurements: pcew, primary chamber external width; scew, secondary chamber external width.

Diplectanids collected from fish gills were prepared by three methods: a) mounted in ammonium picrate-glycerine [37] (designated as ‘p’); b) mounted in Berlese’s fluid [38] (designated as ‘b’); and c) dehydrated in an ethanol series, stained with carmine and permanently mounted in Canada balsam (designated as ‘c’) [39]. Specimens were drawn using an Olympus BH2 microscope equipped with drawing apparatus and DIC optics. The terminology for the sclerotized parts, i.e. the male quadriloculate organ and the vagina follows Justine (2007) [14]. Measurements, in micrometers, were taken with the help of a custom-made transparent rule and are expressed as the mean followed in parentheses by the range, the standard deviation when n≥30, and (n) the number of observations; measurements were taken as in Fig 1. The measurements of the right-hand haptoral hard-parts and left-hand equivalents were pooled. The measurements of the holotype are separated and indicated by ‘h’. Drawings were scanned and redrawn on a computer using Adobe Illustrator. The museum abbreviation used is as follows: MNHN, Muséum National d’Histoire Naturelle, Paris; NHMUK, Natural History Museum, London.

## Results and discussion

### *Pseudorhabdosynochus riouxi* (Oliver, 1986) Kritsky & Beverley-Burton, 1986

Synonym: *Cycloplectanum riouxi* Oliver, 1986.

Type-host: dusky grouper, *Mycteroperca marginata* (Lowe) (Perciformes, Epinephelidae); synonyms: *Epinephelus guaza* (Linnaeus), *E*. *marginatus* (Lowe).

Molecular identification of fish via DNA barcoding: The COI sequences from three specimens from off Tunisia (Table 1) were already identified and published as *M*. *marginata*: KX255749 [22], KU739519, and KU739521 [31].

Site of infection: Gill lamellae.

Type locality: Off Cap Béar (Mediterranean Sea), France.

Other localities: Mediterranean Sea [30, 40, 41]; North Atlantic Ocean [30, 42, 43] (but see text); off Sfax, Tunisia (present paper).

Material examined: Holotype MNHN 26TF-Tj 144; paratype MNHN 27TF-Tj 145; voucher specimens collected by Oliver MNHN HEL68 OLI8-67 to 8–70, voucher specimens newly collected from Tunisia MNHN HEL560, MNHN HEL590.

Prevalence: In our newly collected specimens from Tunisia, 3/3.

#### Redescription (Figs 2 and 3; Table 2)

**Fig 2:**
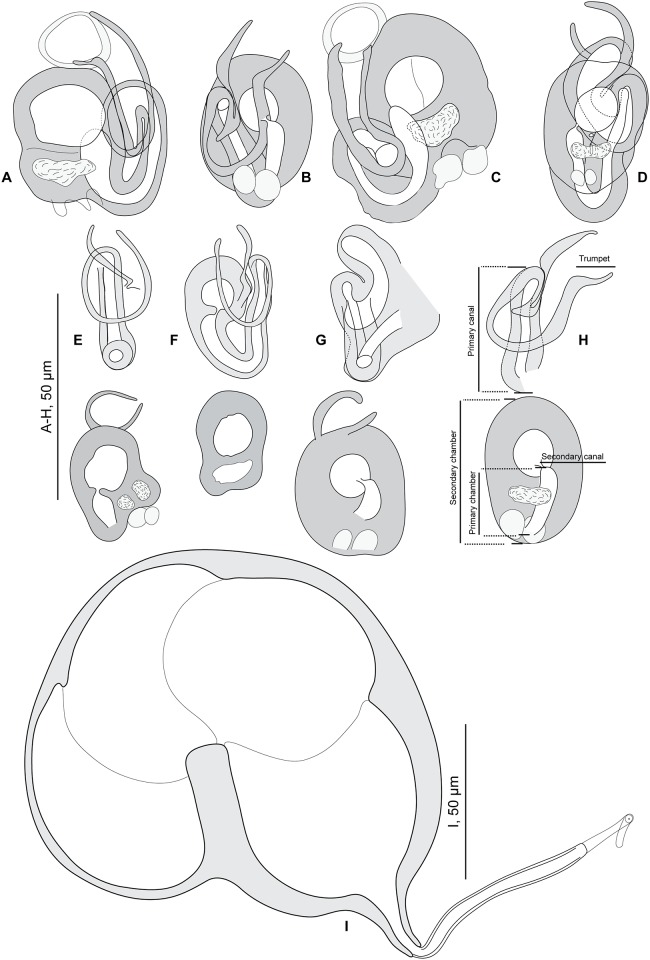
*Pseudorhabdosynochus riouxi* from *Mycteroperca marginata*, quadriloculate organ and various morphologies of vagina. A-D, general structures of vaginae from different specimens, drawn with all parts in a single drawing. A, C, newly collected specimens from Tunisia, MNHN HEL560, MNHN HEL590; B, holotype MNHN 26TF -Tj144 (see also H); D, voucher MNHN HEL68 OLI8-70. E-H, vaginae from different specimens, drawn as two focus planes to show various parts (upper row: canals; lower row: chambers). E, voucher MNHN HEL68 OLI8-68; F, voucher MNHN HEL68 OLI8-69; G, paratype MNHN 27TF-Tj145; H, holotype MNHN 26TF -Tj144. I, quadriloculate male organ, newly collected specimen from Tunisia, MNHN HEL590. A, C, I, Berlese; B, D-H, carmine.

**Fig 3:**
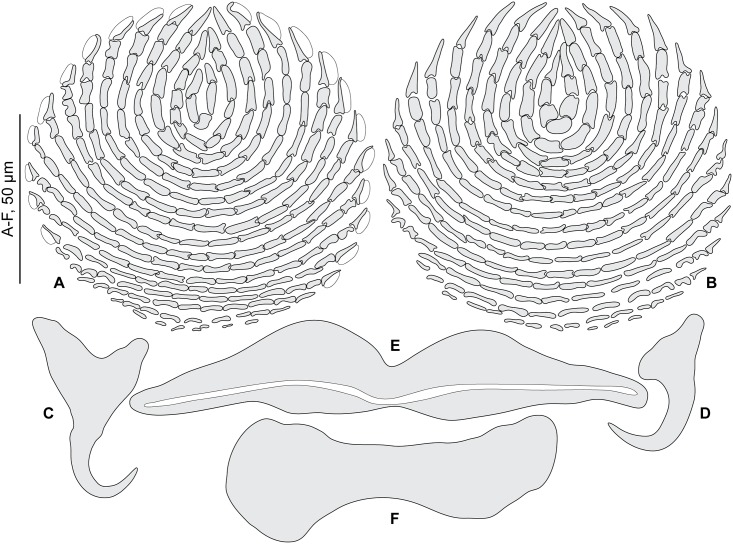
*Pseudorhabdosynochus riouxi* from *Mycteroperca marginata*, squamodiscs and haptoral parts. A, B, squamodiscs (A, dorsal; B, ventral); C-F, haptoral parts (C, ventral anchor; D, dorsal anchor; E, ventral bar; F, lateral bar). A, B, voucher MNHN HEL68 OLI8-70, France. C-F, MNHN HEL590, Tunisia. A-B, carmine; C-F, Berlese.

**Table 2:** *Pseudorhabdosynochus riouxi*, measurements. Means of measurements of sclerotised vaginae, the most important character for systematics, are indicated in bold.

Source	Oliver (1968)	Holotype	Paratype	Vouchers collected by Oliver	Vouchers, newly collected specimens
Registration number		MNHN 26 TF-Tj 144	MNHN 27 TF-Tj 145	MNHN HEL68 OLI 8–67 to 8–70	MNHN HEL560, HEL590
Locality	Off Cap Béar, France (Mediterranean Sea); off Skelligs, Ireland (Atlantic Ocean) *	Off Cap Béar, France (Mediterranean Sea)	Off Cap Béar, France (Mediterranean Sea)	Off Cap Béar, France; off Banyuls-sur-Mer, France (Mediterranean Sea)	Off Sfax, Tunisia (Mediterranean Sea)
Methods	-	Carmine	Carmine	Carmine	Berlese
n	-	-	-	4	5
Body Length	700–800*	304	-	651 (448–864, n = 3)	-
Body Width	350–440*	128	-	363 (240–448, n = 3)	-
Haptor Width	220–270	96	-	184 (144–224, n = 2)	-
Pharynx Length	47–63	65	53	58 (40–75, n = 3)	-
Pharynx Width	41–63	55	58	60 (46–75, n = 4)	-
Penis Internal Length	178–191**	94	87	92 (87–97, n = 2)	105 (100–112, n = 4)
Penis Cone Length	-	18	-	18 (n = 2)	18 (18–19, n = 2)
Penis Tube Length	-	65	32	-	61 (50–72, n = 2)
Penis Tube Diameter	-	5	5	-	5 (4–6, n = 2)
Penis Filament Length	-		**-**	-	-
Sclerotized Vagina Total Length	-	**45**	**44**	**42** (37–53, n = 4)	**52** (47–55, n = 5)
Secondary Chamber External Diameter	-	**26**	**26**	**22** (17–26, n = 4)	**27** (23–34, n = 4)
Squamodisc Length	-	100 (94–106, n = 2)	-	106 (100–112, n = 2)	-
Squamodisc Width	104–106*	97 (94–100, n = 2)	-	119 (119–119, n = 2)	-
Squamodisc, Number of Rows	11–17*	14–15 (n = 2)	-	16–17	-
Squamodisc, Number of Closed Rows	2	2–3	2	2	-
Ventral Anchor Outer Length	47–62	-	-	55 (54–55, n = 2)	59 (57–60, n = 6)
Ventral Anchor Inner Length	-	-	-	45 (40–49, n = 2)	48 (45–52, n = 6)
Dorsal Anchor Outer Length	42–54	43 (n = 2)	-	41 (39–43, n = 2)	43 (41–44, n = 5)
Dorsal Anchor Inner Length	-	25 (n = 2)	-	25 (24–25, n = 2)	22 (20–25, n = 5)
Ventral Bar Length	117–153*	125	-	119 (118–120, n = 2)	154 (153–155, n = 3)
Ventral Bar Width	-	12	-	17 (14–18, n = 3)	26 (22–30, n = 3)
Lateral Bar Length	68–103	75 (n = 2)	-	72 (70–75, n = 6)	100 (96–103, n = 6)
Lateral Bar Width		25 (24–25, n = 2)	-	24 (15–30, n = 6)	37 (31–43, n = 6)

* We consider only the measurements given for specimens from the Mediterranean Sea (Cap Béar)

** “bulbe sclérifié du cirre”, probably refers to penis internal length + cone + tube

Measurements based on 11 specimens in Berlese and carmine; holotype in carmine. Body length h 304, c 564 (304–864, n = 4), including haptor; maximum width h 128, c 304 (128–448, n = 4) at level of ovary. Tegument smooth. Anterior region with 3 pairs of head organs and 2 pairs of dorsal eye-spots, distance between outer margins of anterior eye-spots h 53, b 40 (28–53, n = 4), c 50 (40–62, n = 5), of posterior eye-spots h 62, b 43 (34–62, n = 4), c 52 (43–62, n = 5). Pharynx median, subspherical, length h 65, c 59 (40–75, n = 5), width h 55, c 59 (46–75, n = 6). Haptor bearing two similar squamodiscs, two pairs of lateral anchors, one ventral bar and two lateral (dorsal) bars (Fig 3) and 14 hooklets, width h 96, c 155 (96–224, n = 3). Squamodiscs with 14–17 concentric rows of rodlets; 2 or 3 innermost rows closed (Fig 3A and 3B). Squamodiscs length h 100 (94–106, n = 2), c 106 (100–112, n = 2), width h 97 (94–100, n = 2), c 119 (n = 2). Ventral anchors with distinct handle and guard, outer length b 59 (57–60, n = 6), c 55 (54–55, n = 2), inner length b 48 (45–52, n = 6), c 45 (40–49, n = 2) (Fig 3C). Dorsal anchors with indistinct guard, outer length h 43, b 43 (41–44, n = 5), c 42 (39–43, n = 4), inner length h 25, b 22 (20–25, n = 5), c 25 (24–25, n = 4) (Fig 3D). Lateral (dorsal) bar, with flattened medial end, length h 75, b 100 (96–103, n = 6), c 73 (70–75, n = 8), maximum width h 25, b 37 (31–43, n = 6), c 24 (15–30, n = 8) (Fig 3F). Ventral bar, length h 125, b 154 (153–155, n = 3), c 121 (118–125, n = 3), width h 12, b 26 (22–30, n = 3), c 16 (12–18, n = 4) (Fig 3E). Male copulatory organ a quadriloculate organ, first (anterior) chamber as sclerotized as the three others; fourth chamber forming short cone, prolonged by thin sclerotized tube, inner length h 94, b 105 (100–112, n = 4), c 91 (87–96, n = 4); cone length h 18, b 19 (18–19, n = 2), c 18 (n = 3); tube length h 65, b 61 (50–72, n = 2), c 55 (32–68, n = 3); tube diameter h 5, b 5 (4–6, n = 2), c 5 (n = 3). Filament sometimes observed (Fig 2I).

*Sclerotized vagina* comprises trumpet, primary canal, primary chamber, secondary canal and secondary chamber. Trumpet funnel-shaped, sometimes with "ring-like" distal limit, followed by long primary canal looped in its proximal or medium portion, with thick wall. Primary chamber, elongate with wide lumen, much smaller than secondary chamber that is robust and spherical. Primary and secondary chambers surrounded by very thick and rigid wall. Secondary canal linking the two chambers not always visible (Fig 2A–2H). Total length of the sclerotized vagina h 45, b 52 (47–55, n = 5), c 43 (37–53, n = 6). External diameter of secondary chamber h 26, b 27 (23–34, n = 4), c 23 (17–26, n = 6).

#### Comments

*Pseudorhabdosynochus riouxi* was originally described by Oliver (1986) as *Cycloplectanum riouxi* from *Mycteroperca marginata* in the Mediterranean Sea off France [30]. It was then transferred to *Pseudorhabdosynochus* by Kritsky & Beverley-Burton (1986) [44].

Although all slides were prepared by the same method, “mounted in carmine” (i.e. mounted in Canada balsam after carmine staining), Oliver (1986) mentioned that the specimens of *P*. *riouxi* found in the Mediterranean Sea differed greatly in the measurements from those of the Atlantic Ocean. Yang et al. (2005) subsequently provided a new illustration of *P*. *riouxi* on the basis of eight paratypes (1982.12.14.1–3) deposited in the NHMUK collections by Guy Oliver; these paratypes were collected from the same host, *M*. *marginata*, in the Atlantic Ocean but in a different location, off Ireland’s coast [30, 45].

After examination of the holotype, paratype, and additional voucher specimens deposited in the MNHN collections by Guy Oliver after his retirement, we consider that our newly collected specimens from the same host and from the same region (i.e. the Mediterranean Sea) are conspecific with *P*. *riouxi* (Table 2, Figs 2 and 3). However, they are different from Yang et al.’s drawing by the proportions of vaginal parts and by the total number of concentric rows of rodlets in the squamodiscs (14–17 in the Mediterranean Sea vs. 23–24 in the Atlantic Ocean, as indicated by Oliver, 1986).

It is likely that the species of *Pseudorhabdosynochus* on *M*. *marginata* in the northeastern Atlantic is different from *P*. *riouxi*, and its status would require additional study and collection of new specimens from the same locality. It would also be interesting to know whether the specimens collected off the Canary Islands, mentioned but not described [43], are closer to the Mediterranean species or to the North Atlantic species; the Canary Islands are in the Atlantic but south of the Mediterranean Sea. However, the status of *P*. *riouxi* itself, based on its holotype, which is from the Mediterranean Sea, is unchanged, and specimens from the Mediterranean Sea with the same vaginal structure can safely be attributed to *P*. *riouxi*.

#### Differential diagnosis

*Pseudorhabdosynochus riouxi* most closely resembles *P*. *bouaini* Neifar & Euzet, 2007 and *P*. *enitsuji* Neifar & Euzet, 2007 from the goldblotch grouper *Mycteroperca costae* in the general morphology of the sclerotized vagina. In the three congeners, the sclerotized vagina has a conspicuous spherical secondary chamber. *Pseudorhabdosynochus riouxi* is readily distinguished from *P*. *bouaini* by the length of its primary canal (very long in *P*. *riouxi* vs. very short in *P*. *bouaini*), and by the shape of its primary chamber (more elongate in *P*. *riouxi*). It differs from *P*. *enitsuji* by the size of the vagina (carmine: 37–53 in *P*. *riouxi* vs. 27–34 in *P*. *enitsuji*), by the shape of the primary canal (looped in *P*. *riouxi* vs. roughly straight or curved) and its length (longer in *P*. *riouxi*), and by the external diameter of the secondary chamber (carmine: 17–26 in *P*. *riouxi* vs. 15–18).

A series of species from off the American Atlantic coast show vaginal morphologies that are similar to *P*. *riouxi*, but none is identical. The comparison with these species is given below in the paragraph concerning the’ *P*. *riouxi* group’.

### *Pseudorhabdosynochus bouaini* Neifar & Euzet, 2007

Type-host: Goldblotch grouper, *Mycteroperca costae* (Steindachner) (Perciformes, Epinephelidae); synonyms: *Epinephelus alexandrinus* (Valenciennes), *E*. *costae*.

Molecular identification of fish via DNA barcoding: The COI sequences from four specimens from Tunisia were already published and identified as *M*. *costae* (Table 1).

Site of infection: Gill lamellae.

Type-locality: Off Sfax, Tunisia [24], (off Zarzis, Tunisia on the label).

Other localities: Off Zarzis (Tunisia) [24]; off Dakar (Senegal) [24]; off Tripoli (fish market), Libya (present study).

Material examined: Holotype MNHN HEL1-Th74; paratypes MNHN HEL2-Th75, MNHN HEL3-Th76; voucher specimens collected by Neifar & Euzet MNHN 36HG; voucher specimens newly collected from Tunisia MNHN HEL562; voucher specimens newly collected from Libya MNHN HEL592.

Prevalence: In our specimens from Tunisia, 4/4 (100%); from Libya, 1/1 (100%).

#### Redescription (Figs 4 and 5; Table 3)

**Fig 4:**
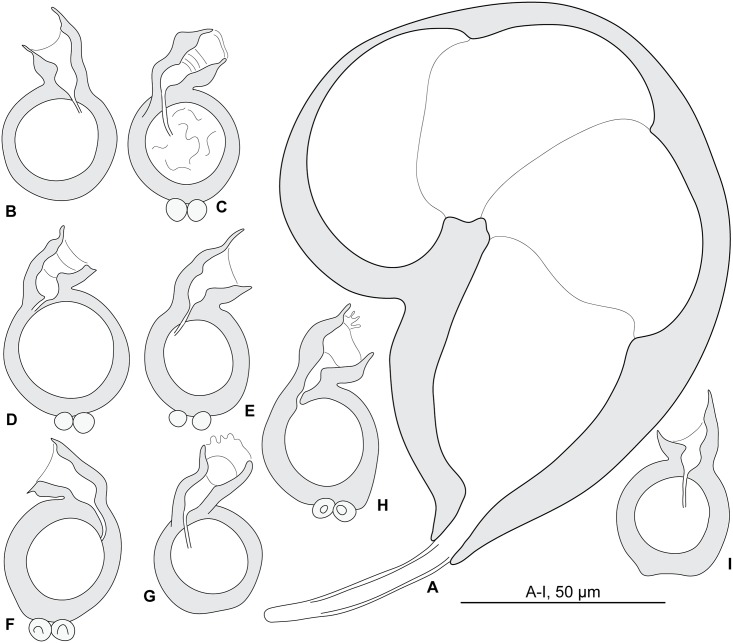
*Pseudorhabdosynochus bouaini* from *Mycteroperca costae*, quadriloculate organ and various morphologies of vagina. A, quadriloculate organ. B-I, vaginae. All, MNHN HEL562, Tunisia, Berlese.

**Fig 5:**
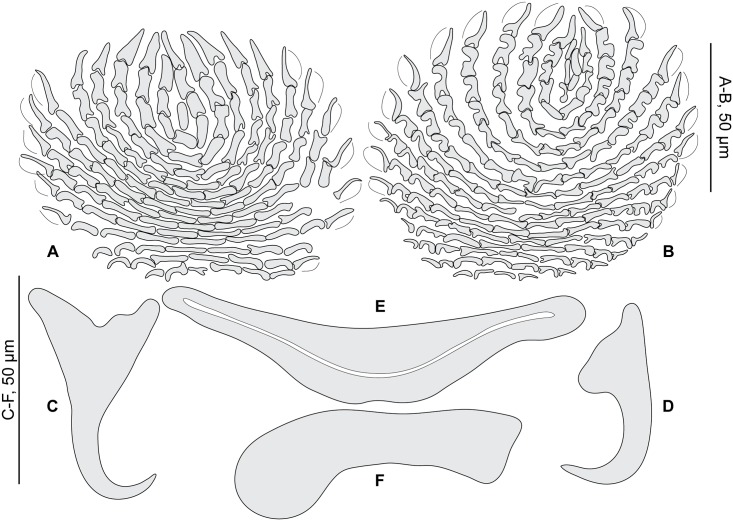
*Pseudorhabdosynochus bouaini* from *Mycteroperca costae*, squamodiscs and haptoral parts. A, B, squamodiscs (A, ventral; B, dorsal). C-F, haptoral parts (C, ventral anchor; D, dorsal anchor; E, ventral bar; F, lateral bar). All, MNHN HEL562, Tunisia, Berlese.

**Table 3:** *Pseudorhabdosynochus bouaini*, measurements. Means of measurements of sclerotised vaginae, the most important character for systematics, are indicated in bold.

Source	Neifar & Euzet, 2007	Holotype	Paratypes	Vouchers collected by Neifar & Euzet	Vouchers, newly collected specimens
Registration number		MNHN HEL1-Th74	MNHN HEL2-Th75, HEL3-Th76	MNHN 36HG	MNHN HEL562
Locality	Off Sfax, off Zarzis, Tunisia (Mediterranean Sea); off Dakar, Senegal (Atlantic Ocean)	Off Zarzis, Tunisia (Mediterranean Sea)	Off Sfax, Tunisia (Mediterranean Sea)	Off Sfax, off Zarzis, Tunisia (Mediterranean Sea)	Off Sfax, Tunisia (Mediterranean Sea)
Methods	Picrate, Berlese, carmine, hemalum-eosin	Picrate	Picrate	Picrate	Berlese
n	16	-	4	4	12
Body Length	1,470 (1,150–1,800, n = 11)	560	1,109 (960–1,344, n = 3)	940 (624–1,088, n = 4)	1,178 (800–1,440, n = 8)
Body Width	390 (300–500, n = 11)	304	240 (192–320, n = 4)	260 (208–320, n = 4)	298 (160–480, n = 11)
Haptor Width	-	176	171 (160–176, n = 3)	192 (176–208, n = 3)	218 (176–304, n = 10)
Pharynx Length	92 (80–120, n = 13)	47	52 (50–56.25, n = 4)	55 (50–65, n = 4)	-
Pharynx Width	75 (65–95, n = 13)	44	51 (50–55, n = 4)	48 (41–53, n = 4)	-
Penis Internal Length	174 (150–190, n = 21)	-	112 (109–115, n = 4)	113 (100–125, n = 4)	129 (112–150, n = 7)
Penis Cone Length	20 (15–25, n = 21)	-	18 (15–20, n = 3)	18 (15–20, n = 2)	16 (10–20, n = 8)
Penis Tube Length	55 (50–65, n = 21)	-	39 (38–40, n = 3)	40 (n = 2)	48 (43–60, n = 9)
Penis Tube Diameter	-	-	5 (4–5, n = 3)	6 (5–6, n = 2)	10 (5–46, n = 9)
Penis Filament Length	-	-	5 (4–5, n = 2)		14 (10–20, n = 5)
Sclerotized Vagina Total Length	**62** (55–75, n = 16)	**40**	**39** (38–40, n = 4)	**40** (37–43, n = 4)	**48** (40–53, n = 12)
Secondary Chamber External Diameter	**35** (30–40, n = 16)	**28**	**25** (24–25, n = 4)	**28** (25–33, n = 4)	**28** (25–30, n = 12)
Squamodisc Length	101 (90–110, n = 8)	-	60 (n = 1)	70 (n = 1)	98 (80–120, n = 10)
Squamodisc Width	109 (100–120, n = 8)	-	-	78 (n = 1)	102 (85–115, n = 10)
Squamodisc, Number of Rows	15 (14–16, n = 9)	-	-	14 (n = 1)	14 (12–16, n = 6)
Squamodisc, Number of Closed Rows	1–2	-	1	1–2	1–2
Ventral Anchor Outer Length	65 (62–70, n = 16)	-	47 (45–49, n = 4)	50 (42–54, n = 4)	52 (48–54, n = 15)
Ventral Anchor Inner Length	-	-	44 (43–46, n = 4)	45 (43–47, n = 5)	44 (40–47, n = 17)
Dorsal Anchor Outer Length	58 (46–62, n = 16)	42 (n = 1)	-	44 (43–44, n = 2)	43 (35–47, n = 19)
Dorsal Anchor Inner Length	-	25 (n = 1)	-	27 (25–28, n = 2)	25 (15–32, n = 19)
Ventral Bar Length	112 (95–130, n = 16)	75	83 (80–85, n = 3)	77 (77–78, n = 3)	111 (96–130, n = 8)
Ventral Bar Width	15 (10–20, n = 16)	15	14 (14–15, n = 3)	15 (14–16, n = 3)	20 (16–22, n = 8)
Lateral Bar Length	80 (75–90, n = 16)	60 (59–61, n = 2)	-	60 (58–62, n = 6)	82 (70–95, n = 21)
Lateral Bar Width	14 (12–15, n = 16)	11 (9–13, n = 2)	-	22 (20–26, n = 6)	25 (15–35, n = 21)

Measurements based on 22 specimens in Berlese and picrate; holotype in picrate. Body length h 560, b 1178 (800–1,440, n = 8), p 956 (560–1,344, n = 8) including haptor; maximum width h 304, b 295 (160–480, n = 12), p 256 (192–320, n = 9) at level of ovary. Tegument smooth. Anterior region with 3 pairs of head organs and 2 pairs of dorsal eye-spots, distance between outer margins of anterior eye-spots b 41 (22–58, n = 12), p 39 (31–47, n = 6), of posterior eye-spots b 37 (22–50, n = 12), p 35 (25–44, n = 7). Pharynx median, subspherical, length h 47, p 53 (47–65, n = 9), width h 44, p 49 (40–55, n = 9). Haptor bearing two similar squamodiscs, two pairs of lateral anchors, one ventral bar and two lateral (dorsal) bars (Fig 5) and 14 hooklets, width h 176, b 218 (176–304, n = 10), p 181 (160–208, n = 7). Squamodiscs with 14–16 concentric rows of rodlets; 1 or 2 innermost rows closed (Fig 5A and 5B). Rodlets sometimes with visible spurs (‘éperons’). Squamodiscs length b 98 (80–120, n = 10), p 65 (60–70, n = 2), width b 102 (85–115, n = 10). Ventral anchors with distinct handle and guard, outer length b 52 (48–54, n = 17), p 48 (42–54, n = 8), inner length b 44 (40–47, n = 19), p 45 (43–47, n = 9) (Fig 5C). Dorsal anchors with indistinct guard, outer length h 42, b 43 (35–47, n = 21), p 43 (42–44, n = 4), inner length h 25, b 25 (15–32, n = 21), p 26 (25–28, n = 4) (Fig 5D). Lateral (dorsal) bar, with flattened medial end, length h 60, b 80 (60–95, n = 23), p 60 (58–62, n = 14), maximum width h 11, b 25 (15–35, n = 23), p 19 (9–26, n = 14) (Fig 5F). Ventral bar, length h 75, b 108 (82–130, n = 9), p 79 (75–85, n = 7), width h 15, b 19 (12–22, n = 9), p 15 (14–16, n = 7) (Fig 5E). Male copulatory organ a quadriloculate organ (Fig 4A), first (anterior) chamber as sclerotized as the three others; fourth chamber forming short cone, prolonged by thin sclerotized tube, inner length b 130 (112–150, n = 8), p 112 (100–125, n = 8); cone length b 16 (10–20, n = 9), p 18 (15–20, n = 5); tube length b 47 (41–60, n = 10), p 40 (38–40, n = 5); tube diameter b 6 (5–6, n = 10), p 5 (4–6, n = 5); end of tube prolonged by thin unsclerotised filament, length p 4 (4–5, n = 3).

*Sclerotized vagina* comprises anterior trumpet followed by primary canal; primary canal heavily sclerotized, very short, roughly straight, its lumen wide; connection between primary canal and primary chamber anterior; primary chamber very similar in wall thickness to primary canal, heavily sclerotized, ovate with wide lumen; secondary canal inserted at anterior limit of secondary chamber, well visible, its lumen thin; secondary chamber robust, spherical, much larger than primary chamber (Fig 4B–4I). Total length of sclerotized vagina h 40, b 47 (40–53, n = 13), p 40 (37–43, n = 9). External diameter of secondary chamber h 28, b 27 (25–30, n = 13), p 26 (24–33, n = 9).

#### Comments

The type-locality of *Pseudorhabdosynochus bouaini* (off Sfax, Tunisia) reported by Neifar & Euzet in the original description is different from that indicated on the label of the type-slide (off Zarzis, Tunisia). Under Recommendation 76A.2 of the International Code of Zoological Nomenclature [46], the type-locality of *P*. *bouaini* should be corrected from “off Sfax” to “off Zarzis”.

In the slides containing the paratypes MNHN HEL2-Th75 and MNHN voucher specimens labelled as *P*. *bouaini*, additional specimens of *P*. *enitsuji* were found. The two species co-occur on the gills of *M*. *costae* in the Mediterranean Sea and eastern Atlantic Ocean.

#### Differential diagnosis

Just like *P*. *bouaini*, *P*. *enitsuji* and *P*. *riouxi* possess a sclerotized vagina with a conspicuous spherical secondary chamber. Among species from the Mediterranean Sea, *Pseudorhabdosynochus bouaini* is closest morphologically to *P*. *enitsuji*. The two species can be differentiated by the size of the vagina, i.e. larger vagina in *P*. *bouaini* (picrate: 40 vs. 33; Berlese: 48 vs. 39), the length of the primary canal (shorter in *P*. *bouaini*), and the external diameter of the secondary chamber (picrate: 28 in *P*. *bouaini* vs. 19; Berlese: 28 in *P*. *bouaini* vs. 23). *Pseudorhabdosynochus bouaini* is readily distinguished from *P*. *riouxi* by its very short primary canal (vs. very long and looped in *P*. *riouxi*) and by its very small primary chamber.

A series of species from off the American Atlantic coast show vaginal morphologies that are similar to *P*. *bouaini*, but none is identical. The comparison with these species is given below in the paragraph concerning the ‘*P*. *riouxi* group’.

### *Pseudorhabdosynochus enitsuji* Neifar & Euzet, 2007

Type-host: The goldblotch grouper, *Mycteroperca costae* (Steindachner) (Perciformes, Epinephelidae); synonyms: *Epinephelus alexandrinus* (Valenciennes), *E*. *costae*.

Molecular identification of fish via DNA barcoding: The COI sequences from four specimens from Tunisia were already published and identified as *M*. *costae* (Table 1).

Site of infection: Gill lamellae.

Type-locality: Off Sfax, Tunisia [24].

Other localities: Off Zarzis (Tunisia) [24]; off Dakar (Senegal) [24]; off Tripoli (fish market), Libya (present study).

Material examined: Holotype MNHN HEL7-Th80 (3 specimens on 1 slide, see below); paratypes MNHN HEL8 Th81, MNHN HEL9 Th82; voucher specimens collected by Justine, MNHN 36HG; voucher specimens collected by Neifar & Euzet MNHN 36HG; voucher specimens newly collected from Tunisia MNHN HEL562; voucher specimens newly collected from Libya MNHN HEL592.

Designation of a neotype MNHN HEL7-Th80: See below.

Prevalence: In our specimens from Tunisia, 4/4 (100%); from Libya, 1/1 (100%).

#### Redescription (Figs 6–8; Table 4)

**Fig 6:**
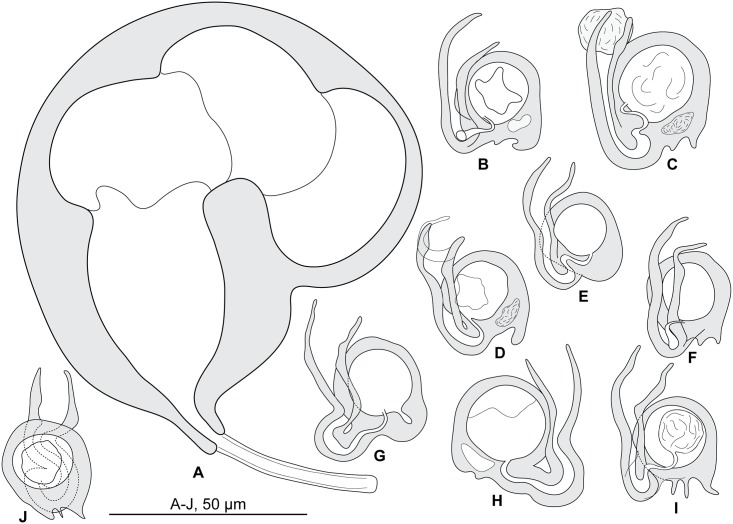
*Pseudorhabdosynochus enitsuji* from *Mycteroperca costae*, quadriloculate organ and various morphologies of vagina. A, quadriloculate organ; B-J, vaginae. A, B-D, G-J, Tunisia, MNHN HEL562; E-F, Tunisia, MNHN 36HG (deposited by Neifar & Euzet). A-D, G-J, Berlese; E-F, picrate.

**Fig 7:**
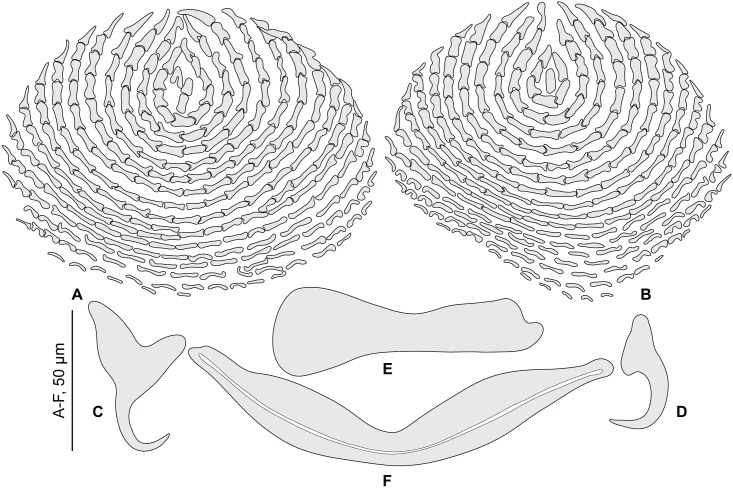
*Pseudorhabdosynochus enitsuji* from *Mycteroperca costae*, squamodiscs and haptoral parts. A, B, squamodiscs (A, ventral; B, dorsal). C-F, haptoral parts (C, ventral anchor; D, dorsal anchor; E, lateral bar; F, ventral bar). All, MNHN HEL562, Tunisia, Berlese.

**Fig 8:**
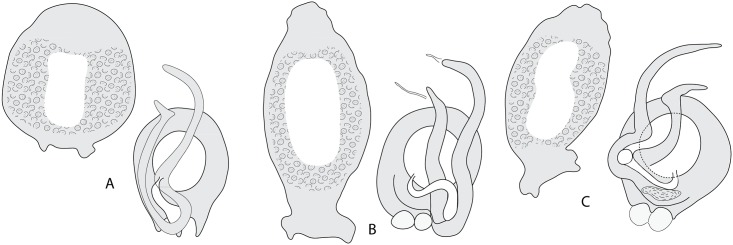
The three specimens found in the type-slide of *Pseudorhabdosynochus enitsuji*. A, B, C, left, habitus, right, sclerotized vagina. All, carmine. Specimen B is designated here as the neotype of the species.

**Table 4:** *Pseudorhabdosynochus enitsuji*, measurements. Means of measurements of sclerotised vaginae, the most important character for systematics, are indicated in bold.

Source	Neifar & Euzet, 2007	Neotype	Paratypes	Vouchers collected by Justine	Vouchers collected by Neifar & Euzet	Vouchers, specimens newly collected
Registration number		MNHN HEL7 Th80	MNHN HEL8-Th81, HEL9-Th 82	MNHN 36HG	MNHN 36HG	MNHN HEL562
Locality	Off Sfax, off Zarzis, Tunisia (Mediterranean Sea); off Dakar, Senegal (Atlantic Ocean)	Off Sfax, Tunisia (Mediterranean Sea)	Off Sfax, Tunisia (Mediterranean Sea)	Off Dakar, Senegal (Atlantic Ocean)	Off Sfax, Tunisia (Mediterranean Sea)	Off Sfax, Tunisia (Mediterranean Sea)
Methods	Picrate, Berlese, carmine, hemalum-eosin	Carmine	Carmine	Carmine	Picrate	Berlese
n	19	-	5	16	3	9
Body Length	1,390 (1,100–1,650, n = 11)	608*	512 (416–608, n = 5)	693 (416–1,280, n = 15)	1,083 (896–1,312, n = 3)	1,120 (784–1,424, n = 8)
Body Width	460 (300–650, n = 11)	256*	304 (256–336, n = 5)	289 (192–336, n = 15)	336 (320–352, n = 3)	525 (368–800, n = 6)
Haptor Width	-	176*	160 (144–176, n = 2)	190 (160–240, n = 8)	208 (176–240, n = 3)	256 (256–256, n = 3)
Pharynx Length	88 (80–100, n = 17)	50*	54 (48–62, n = 4)	56 (40–75, n = 15)	66 (62–69, n = 3)	83 (62–100, n = 3)
Pharynx Width	76 (60–85, n = 17)	40*	48 (44–50, n = 4)	51 (40–58, n = 15)	57 (50–61, n = 3)	61 (37–82, n = 3)
Penis Internal Length	155 (140–210, n = 16)	45*	55 (n = 3)	84 (60–137, n = 16)	-	114 (95–137, n = 4)
Penis Cone Length	12 (10–15, n = 16)	8*	10 (7–11, n = 3)	12 (7–18, n = 14)	14 (12–16, n = 3)	20 (n = 2)
Penis Tube Length	58 (55–70, n = 16)	40*	39 (38–40, n = 3)	41 (36–45, n = 14)	43 (41–45, n = 3)	47 (40–63, n = 5)
Penis Tube Diameter	-	3*	4 (3–5, n = 4)	4 (3–5, n = 16)	5 (3–5, n = 3)	5 (n = 5)
Penis Filament Length	-	6*	-	24 (6–41, n = 2)	-	-
Sclerotized Vagina Total Length	**42** (40–45, n = 19)	25*	**31** (27–34, n = 5)	**32** (29–35, n = 16)	**33** (30–36, n = 3)	**39** (35–45, n = 11)
Secondary Chamber External Diameter	**27** (25–30, n = 19)	**17**	**17** (15–18, n = 5)	**19** (16–20, n = 16)	**19** (17–21, n = 3)	**23** (21–25, n = 11)
Squamodisc Length	105 (100–120, n = 7)	61 (n = 1) *	63 (60–65, n = 2)	64 (54–75, n = 18)	80 (76–83, n = 4)	95 (72–157, n = 12)
Squamodisc Width	94 (90–100, n = 7)	67 (n = 1) *	74 (67–80, n = 2)	73 (45–89, n = 18)	89 (83–94, n = 4)	109 (90–144, n = 12)
Squamodisc, Number of Rows	15 (14–16, n = 7)	16 (n = 1) *	15 (n = 1)	15–18	15 (n = 4)	14–17
Squamodisc, Number of Closed Rows	3–4	2 (n = 1) *	2 (n = 1)	1–3	3 (n = 4)	1–3
Ventral Anchor Outer Length	66 (62–72, n = 14)	-	-	45 (34–52, n = 13)	49 (46–52, n = 4)	51 (37–57, n = 17)
Ventral Anchor Inner Length	-	-	40 (39–40, n = 2)	37 (30–43, n = 14)	40 (38–41, n = 4)	39 (31–44, n = 17)
Dorsal Anchor Outer Length	50 (45–52, n = 14)	-	32 (28–35, n = 4)	35 (31–38, n = 20)	37 (36–38, n = 4)	39 (37–41, n = 19)
Dorsal Anchor Inner Length	-	-	19 (16–21, n = 3)	22 (19–25, n = 20)	24 (22–25, n = 4)	23 (20–25, n = 19)
Ventral Bar Length	138 (130–160, n = 16)	108*	93 (85–97, n = 3)	95 (90–99, n = 15)	111 (105–118, n = 3)	151 (132–183, n = 11)
Ventral Bar Width	15 (10–17, n = 16)	14*	12 (10–15, n = 3)	12 (8–15, n = 15)	15 (12–17, n = 3)	21 (14–25, n = 10)
Lateral Bar Length	80 (70–85, n = 14)	55 (n = 2) *	54 (52–57, n = 8)	53 (49–58, n = 30)	64 (62–65, n = 6)	85 (75–97, n = 22)
Lateral Bar Width	14 (12–17, n = 14)	15 (14–15, n = 2)*	15 (10–21, n = 5)	17 (8–23, n = 3)	21 (18–24, n = 6)	28 (10–39, n = 22)

* Neotype designated in this paper, among three specimens on the type-slide.

Measurements based on 40 specimens in Berlese, picrate and carmine (including the neotype in carmine). Body length 608, b 1,120 (784–1,424, n = 8), p 1,083 (896–1,312, n = 3), c 603 (304–1,280, n = 25), including haptor; maximum width 256, b 525 (368–800, n = 6), p 336 (320–352, n = 3), c 292 (192–336, n = 25) at level of ovary. Tegument smooth. Anterior region with 3 pairs of head organs and 2 pairs of dorsal eye-spots, distance between outer margins of anterior eye-spots b 69 (37–100, n = 6), p 48 (37–53, n = 3), c 39 (33–46, n = 14), of posterior eye-spots b 62 (48–100, n = 7), p 50 (40–56, n = 3), c 40 (34–44, n = 14). Pharynx median, subspherical, length 50, b 83 (62–100, n = 3), p 66 (62–69, n = 3), c 52 (31–75, n = 23), width 40, b 61 (37–82, n = 3), p 57 (50–61, n = 3), c 48 (30–58, n = 23). Haptor bearing two similar squamodiscs, two pairs of lateral anchors, one ventral bar and two lateral (dorsal) bars (Fig 7) and 14 hooklets, width b 256 (n = 3), p 208 (176–240, n = 3), c 181 (144–240, n = 13). Squamodiscs with 14–17 concentric rows of rodlets; 1–3 innermost rows closed (Fig 7A and 7B). Squamodiscs length 61 (n = 1), b 95 (72–157, n = 12), p 80 (76–83, n = 4), c 62 (46–75, n = 26), width 67 (n = 1), b 109 (90–144, n = 12), p 89 (83–94, n = 4), c 72 (45–89, n = 26). Ventral anchors with distinct handle and guard, outer length b 51 (37–57, n = 17), p 49 (46–52, n = 4), c 45 (34–52, n = 13), inner length b 39 (31–44, n = 17), p 40 (38–41, n = 4), c 37 (30–43, n = 16) (Fig 7C). Dorsal anchors with indistinct guard, outer length b 39 (37–41, n = 19), p 37 (36–38, n = 4), c 35 (28–38, n = 26), inner length b 23 (20–25, n = 19), p 24 (22–25, n = 4), c 21 (16–25, n = 25) (Fig 7D). Lateral (dorsal) bar, with flattened medial end, length 55 (n = 2), b 85 (75–97, n = 22), p 64 (62–65, n = 6), c 54±2 (49–58, n = 48), maximum width 15 (14–15, n = 2), b 28 (10–39, n = 22), p 21 (18–24, n = 6), c 17±3.9 (8–24, n = 45) (Fig 7E). Ventral bar, length 108, b 151 (132–183, n = 11), p 111 (105–118, n = 3), c 95 (85–108, n = 23), width 14, b 21 (14–25, n = 10), p 15 (12–17, n = 3), c 13 (8–16, n = 22) (Fig 7F). Male copulatory organ a quadriloculate organ (Fig 6A), first (anterior) chamber as sclerotized as the three others; fourth chamber forming short cone, prolonged by thin sclerotized tube, inner length 45, b 114 (95–137, n = 4), c 77 (45–137, n = 21); cone length 8, b 20 (n = 2), p 14 (12–16, n = 3), c 11 (7–18, n = 18); tube length 40, b 47 (40–63, n = 5), p 43 (41–45, n = 3), c 40 (35–45, n = 19); tube diameter 3, b 5 (n = 5), p 5 (3–5, n = 3), c 4 (3–5, n = 23); end of tube prolonged by thin unsclerotised filament, length 6, c 20 (6–41, n = 4).

*Sclerotized vagina* comprises trumpet funnel-shaped, primary canal heavily sclerotized, roughly straight; followed by curved primary chamber; secondary canal opening into distal limit of secondary chamber, secondary chamber robust and spherical, with thick wall, much larger than primary chamber (Figs 6B–6J, 8). Total length of sclerotized vagina 25, b 39 (35–45, n = 11), p 33 (30–36, n = 3), c 31 (25–35, n = 26). External diameter of secondary chamber 17, b 23 (21–25, n = 11), p 19 (17–21, n = 3), c 18 (15–20, n = 26).

#### Nomenclatural comments

The type-slide of *Pseudorhabdosynochus enitsuji* deposited by Neifar & Euzet in the MNHN collections contains more than one specimen of *P*. *enitsuji* (3 specimens) among which the holotype was not distinctly marked. Neifar & Euzet (2007) provided a composite drawing of this species but the holotype was not represented. We consider that this is a case, although atypical, in which the holotype is “lost or destroyed” since it is impossible to recognize it among several specimens. According to Article 75.3 of the Code [46], the specimen labelled B in our Fig 8 is designated here as the neotype of *P*. *enitsuji*.

#### Differential diagnosis

*Pseudorhabdosynochus enitsuji* is close to *P*. *bouaini* and *P*. *riouxi* in terms of general structure of sclerotized parts, i.e. male copulatory organ, squamodiscs, haptoral parts and mainly the sclerotized vagina. This species is distinguished from its two congeners by having the smaller size of vagina and secondary chamber (more details above in *P*. *riouxi* and *P*. *bouaini*).

A series of species from the American Atlantic coast show vaginal morphologies that are similar to *P*. *enitsuji*, but none is identical. The comparison with these species is given below in the paragraph concerning the’ *P*. *riouxi* group’.

### Proposal for the ‘*Pseudorhabdosynochus riouxi* group’ on *Mycteroperca* spp.

**Fig 9:**
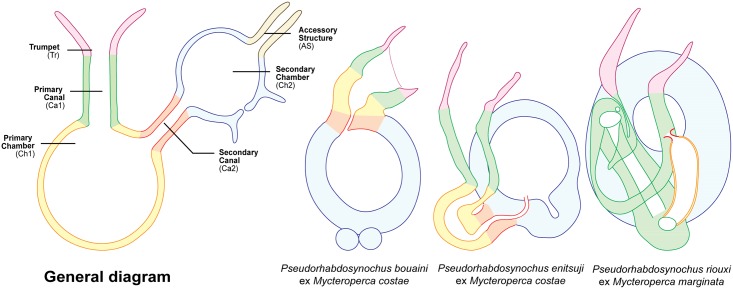
Homologies of the various parts of the sclerotized vaginae of species of the ‘*Pseudorhabdosynochus riouxi* group‘, illustrated by coloured diagrams. The same colours are used in each diagram for the same parts, to show homologies between species. The nomenclature of vaginal parts and the general diagram are from Justine (2007) [14]. All vaginae drawn with similar sizes—magnifications vary.

Among the Mediterranean species of *Pseudorhabdosynochus* infesting the gills of members of *Mycteroperca*, only *P*. *riouxi*, *P*. *bouaini*, and *P*. *enitsuji* share the common characteristic of a sclerotized vagina with a conspicuous and heavily sclerotized spherical secondary chamber (Fig 9). In order to facilitate their distinction within *Pseudorhabdosynochus*, which currently includes more than 80 valid species described on groupers throughout the world, the ‘*P*. *riouxi* group’ is proposed here to accommodate them.

Species of the ‘*P*. *riouxi* group’ are well differentiated from their congeners in the Mediterranean Sea, but the fact that several species of *Pseudorhabdosynochus* may occur on both sides of the Atlantic [21, 25] requires a comparison with species from the western side of the Atlantic.

Six species, found on *Mycteroperca* spp. off the American Atlantic coast, share a single heavily sclerotized spherical chamber with the ‘*P*. *riouxi* group’: *P*. *capurroi* Vidal-Martinez & Mendoza-Franco, 1998 (from *M*. *bonaci*), *P*. *contubernalis* Kritsky, Bakenhaster & Adams, 2015 (from *M*. *phenax*), *P*. *hyphessometochus* Kritsky, Bakenhaster & Adams, 2015 (from *M*. *interstitialis*), *P*. *kritskyi* Dyer, Williams & Bunkley-Williams, 1995 (from *M*. *microlepis*), *P*. *mycteropercae* Kritsky, Bakenhaster & Adams, 2015 (from *M*. *tigris*) and *P*. *vascellum* Kritsky, Bakenhaster & Adams, 2015 (from *M*. *phenax*) [25, 47, 48]. Indeed, Kritsky et al. ([25] p. 13) remarked that groupers assigned to *Mycteroperca* are parasitized by “a complex of similar species”, which have in common a sclerotized vagina “with a single subspherical to ovate chamber and a distal tube that is strongly recurved near its articulation with the vaginal vestibule”. Unfortunately, the descriptions or redescriptions of these species did not include a detailed interpretation of the canals and chambers of the vagina and it is not ascertained whether the “single subspherical to ovate chamber” is really the secondary chamber, as in the Mediterranean species of the ‘*P*. *riouxi* group’. These species also have in common with ‘the *P*. *riouxi* group’ the characteristic of squamodiscs with closed internal rows [25, 48]. We consider it likely that they are close to the ‘*P*. *riouxi* group‘.

None of the above six species have vaginal morphology identical to the three species of the “*P*. *riouxi* group” in the Mediterranean Sea. Differentiation on other characters is as follows:

*P*. *capurroi* has characteristic “twisted” lateral bars [25, 48].

*P*. *contubernalis* has an male copulatory organ (MCO) with a very thin-walled first chamber and tegumental scales on the peduncle [25].

*P*. *kritskyi* has an MCO with a very short cone and dorsal bars with a large median end [25].

*P*. *vascellum* has an MCO with a delicate, almost non-existent cone [25].

The “*P*. *riouxi* group” could be extended to these six species and thus could include nine species; we prefer to keep only the Mediterranean species within this group and wait for additional molecular information to ascertain relationships of the “*P*. *riouxi* group” to species off the American Atlantic coast.

### *Pseudorhabdosynochus dolicocolpos* Neifar & Euzet, 2007

Type-host: The goldblotch grouper, *Mycteroperca costae* (Steindachner) (Perciformes, Epinephelidae); synonyms: *Epinephelus alexandrinus* (Valenciennes), *E*. *costae*.

Molecular identification of fish via DNA barcoding: The COI sequences from four specimens from Tunisia were already published and identified as *M*. *costae* (Table 1).

Site of infection: Gill lamellae.

Type-locality: Off Sfax, Tunisia [24].

Other localities: Off Zarzis (Tunisia) [24]; off Dakar (Senegal) [24]; off Tripoli (fish market), Libya (present study).

Material examined: Holotype MNHN HEL4-Th77 (darkened picrate slide); paratypes MNHN HEL5-Th78, MNHN HEL6-Th79; voucher specimens collected by Neifar & Euzet MNHN 36HG, voucher specimens newly collected from Tunisia MNHN HEL562; voucher specimens newly collected from Libya MNHN HEL592.

Prevalence: In our specimens from Tunisia, 4/4 (100%); from Libya, 1/1 (100%).

#### Redescription (Figs 10 and 11; Table 5)

**Fig 10:**
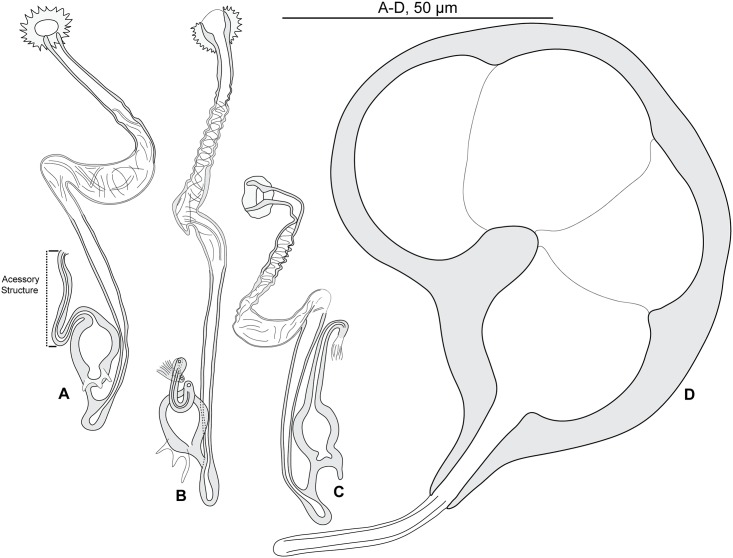
*Pseudorhabdosynochus dolicocolpos* from *Mycteroperca costae*, quadriloculate organ and various morphologies of vagina. A-C, vaginae; D, quadriloculate organ. All, MNHN HEL562, Tunisia, Berlese.

**Fig 11:**
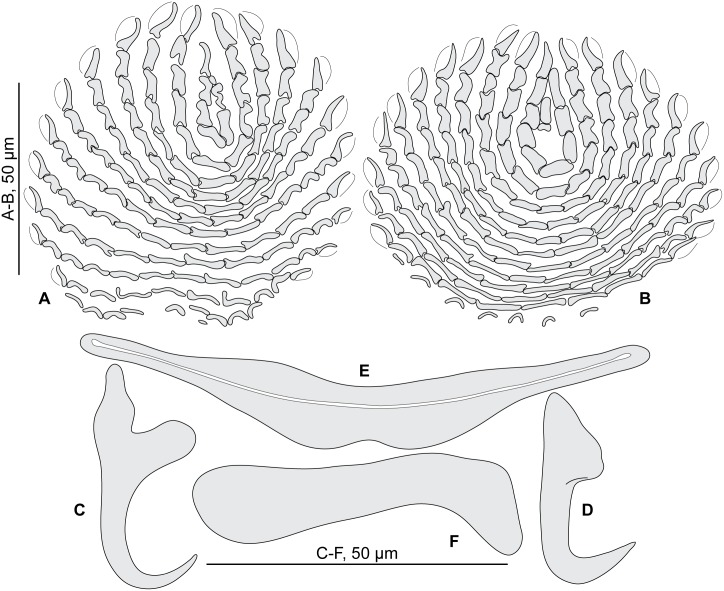
*Pseudorhabdosynochus dolicocolpos* from *Mycteroperca costae*, squamodiscs and haptoral parts. A, B, squamodiscs (A, ventral; B, dorsal); C-F, haptoral parts (C, ventral anchor; D, dorsal anchor; E, ventral bar; F, lateral bar). A, B, MNHN HEL562, Tunisia; C-F, paratype MNHN HEL5-Th78, France. A, B, Berlese; C-F, picrate.

**Table 5:** *Pseudorhabdosynochus dolicocolpos*, measurements. Means of measurements of sclerotised vaginae, the most important character for systematics, are indicated in bold.

Source	Neifar & Euzet, 2007	Paratypes	Vouchers collected by Neifar & Euzet	Vouchers, newly collected specimens
Registration number		MNHN HEL5-Th78, HEL6-Th79	MNHN 36HG	MNHN HEL562
Locality	Off Sfax, off Zarzis, Tunisia (Mediterranean Sea); off Dakar, Senegal (Atlantic Ocean)	Off Sfax, Tunisia (Mediterranean Sea)	Off Sfax, Tunisia (Mediterranean Sea)	Off Sfax, Tunisia (Mediterranean Sea)
Methods	Picrate, Berlese, carmine, hemalum-eosin	Picrate	Picrate	Berlese
n	30	9	7	8
Body Length	790 (650–950, n = 25)	521 (416–640, n = 9)	514 (384–720, n = 7)	672 (608–768, n = 6)
Body Width	267 (180–350, n = 25)	225 (160–416, n = 9)	254 (208–344, n = 5)	306 (240–360, n = 5)
Haptor Width	-	168 (144–192, n = 8)	181 (176–192, n = 7)	190 (176–200, n = 4)
Pharynx Length	62 (52–80, n = 11)	36 (30–38, n = 8)	33 (28–40, n = 7)	-
Pharynx Width	48 (45–50, n = 11)	37 (31–44, n = 8)	36 (28–53, n = 7)	-
Penis Internal Length	98 (75–120, n = 15)	-	-	70 (62–78, n = 7)
Penis Cone Length	21 (17–25, n = 15)	14 (7–18, n = 9)	13 (7–18, n = 6)	16 (12–24, n = 4)
Penis Tube Length	41 (35–45, n = 15)	31 (26–35, n = 9)	31 (30–33, n = 6)	32 (30–35, n = 4)
Penis Tube Diameter	-	3 (n = 9)	3 (3–4, n = 6)	3 (3–4, n = 4)
Penis Filament Length	-	30 (20–44, n = 3)		27 (9–41, n = 3)
Sclerotized Vagina Total Length	**97** (75–120, n = 16)	**62** (55–72, n = 7)	**61** (43–75, n = 6)	**74** (55–92, n = 8)
Primary Chamber External Diameter	-	**3** (3–4, n = 8)	**3** (3–4, n = 6)	**3** (3–4, n = 7)
Secondary Chamber External Diameter	**12** (10–13, n = 16)	-	-	-
Squamodisc Length	-	63 (55–74, n = 11)	64 (56–70, n = 10)	76 (66–95, n = 12)
Squamodisc Width	93 (80–110, n = 14)	76 (60–88, n = 11)	76 (70–82, n = 10)	92 (75–100, n = 12)
Squamodisc, Number of Rows	12–13	8–13	11–12	10–12
Squamodisc, Number of Closed Rows	1–2	1–2	1–2	1
Ventral Anchor Outer Length	53 (50–55, n = 22)	39 (35–43, n = 16)	40 (37–42, n = 7)	40 (37–42, n = 12)
Ventral Anchor Inner Length		30 (25–35, n = 16)	30 (28–32, n = 7)	30 (25–34, n = 12)
Dorsal Anchor Outer Length	43 (36–47, n = 20)	33 (31–34, n = 17)	33 (31–34, n = 6)	32 (26–35, n = 9)
Dorsal Anchor Inner Length	-	18 (17–20, n = 17)	19 (17–21, n = 6)	19 (15–20, n = 9)
Ventral Bar Length	112 (93–125, n = 21)	94 (87–98, n = 9)	91 (82–99, n = 7)	113 (107–125, n = 7)
Ventral Bar Width	14 (10–18, n = 1)	11 (5–14, n = 9)	13 (12–15, n = 7)	15 (10–18, n = 7)
Lateral Bar Length	71 (60–80, n = 20)	56 (49–60, n = 16)	58 (56–62, n = 12)	72 (61–76, n = 14)
Lateral Bar Width	15 (10–20, n = 20)	17 (12–23, = 16)	18 (12–23, n = 12)	21 (15–27, n = 14)

Measurements based on 24 specimens in Berlese and picrate. Body length b 672 (608–768, n = 6), p 324 (240–450, n = 16), including haptor; maximum width b 306 (240–360, n = 5), p 147 (100–260, n = 14) at level of ovary. Tegument smooth. Anterior region with 3 pairs of head organs and 2 pairs of dorsal eye-spots, distance between outer margins of anterior eye-spots b 40 (37–44, n = 6), p 30 (28–37, n = 13), of posterior eye-spots b 37 (31–40, n = 6), p 29 (25–32, n = 13). Pharynx median, subspherical, length p 34 (28–40, n = 15), width p 36 (28–53, n = 15). Haptor bearing two similar squamodiscs, two pairs of lateral anchors, one ventral bar and two lateral (dorsal) bars (Fig 11) and 14 hooklets, width b 190 (176–200, n = 4), p 102 (20–120, n = 15). Squamodiscs with 10–13 concentric rows of rodlets; 1–2 innermost rows closed (Fig 11A and 11B). Rodlets sometimes with visible spurs (‘éperons’). Squamodiscs length b 76 (66–95, n = 12), p 63 (55–74, n = 21), width b 92 (75–100, n = 12), p 76 (60–88, n = 21). Ventral anchors with distinct handle and guard, outer length b 40 (37–42, n = 12), p 39 (35–43, n = 23), inner length b 30 (25–34, n = 12), p 30 (25–35, n = 23) (Fig 11C). Dorsal anchors with indistinct guard, outer length b 32 (26–35, n = 9), p 33 (31–34, n = 23), inner length b 19 (15–20, n = 9), p 19 (17–21, n = 23) (Fig 11D). Lateral (dorsal) bar, with flattened medial end, length b 72 (61–76, n = 14), p 57 (49–62, n = 28), maximum width b 21 (15–27, n = 14), p 17 (12–23, n = 28) (Fig 11F). Ventral bar, length b 113 (107–125, n = 7), p 92 (82–99, n = 16), maximum width, b 15 (10–18, n = 7), p 12 (5–15, n = 16) (Fig 11E). Male copulatory organ a quadriloculate organ (Fig 10D), first (anterior) chamber as sclerotized as the three others; fourth chamber forming short cone, prolonged by thin sclerotized tube, inner length b 70 (62–78, n = 7); cone length b 16 (12–24, n = 4), p 14 (7–18, n = 15); tube length b 32 (30–35, n = 4), p 31 (26–35, n = 15); tube diameter b 3 (3–4, n = 4), p 3 (3–4, n = 15); end of tube prolonged by thin unsclerotised filament, length b 27 (9–41, n = 3), p 29 (20–44, n = 4).

*Sclerotized vagina* comprises anterior trumpet, primary canal, primary chamber, secondary canal, secondary chamber and accessory structure clearly visible. Anterior trumpet sclerotized, ring-shaped, followed by primary canal; primary canal very long, its shape variable, twisted or curved, rarely straight; primary canal thin-walled with irregular diameter, less sclerotized in its medium portion; primary chamber very small, heavily sclerotized; secondary canal originates from proximal portion of primary chamber, slightly sclerotized; secondary chamber roughly spherical, heavily sclerotized, much larger than primary chamber; accessory structure long and robust, emerges from anterior part of secondary chamber (Fig 10A–10C). Total length of sclerotized vagina b 74 (55–92, n = 8), p 62 (43–75, n = 13). External diameter of primary chamber b 3 (3–4, n = 7), p 3 (3–4, n = 14).

#### Differential diagnosis

No species of *Pseudorhabdosynochus* in the Mediterranean Sea shows a sclerotized vagina similar to that of *P*. *dolicocolpos*. *Pseudorhabdosynochus dolicocolpos* is readily distinguished from the species of the ‘*P*. *riouxi* group’ and all other Mediterranean species of *Pseudorhabdosynochus* by its very long primary canal and the general structure of its sclerotized vagina. This species has a ring-shaped trumpet, a character also found in the ‘beverleyburtonae group’ [22]; however, *P*. *dolicocolpos* can be differentiated from the species of this group by the general structure of the rest of the vagina.

Of the described species not from the Mediterranean Sea, a single species has vaginal morphology resembling that of *P*. *dolicocolpos*: *P*. *variabilis* Justine, 2008 from *M*. *morrhua* off New Caledonia [27]. Both species also share similar shapes of haptoral parts. However, *P*. *variabilis* has an MCO with a thin-walled first chamber (vs. thick in *P*. *dolicocolpos*), a relatively longer secondary canal and a shorter sclerotized vagina (c. 50 vs. 75–120 μm), and squamodiscs with 2–3 circular rows (vs. 1–2 V-shaped rows). It is, however, striking that the two species of *Pseudorhabdosynochus* with this vaginal morphology are both parasites of species of *Mycteroperca*, although widely separated geographically (Mediterranean Sea vs. South Pacific). A common origin is likely.

### *Pseudorhabdosynochus sinediscus* Neifar & Euzet, 2007

Type-host: The goldblotch grouper, *Mycteroperca costae* (Steindachner) (Perciformes, Epinephelidae); synonyms: *Epinephelus alexandrinus* (Valenciennes), *E*. *costae*.

Molecular identification of fish via DNA barcoding: The COI sequences from four specimens from Tunisia were already published and identified as *M*. *costae* (Table 1).

Site of infection: Gill lamellae.

Type-locality: Off Sfax, Tunisia.

Other localities: Off Zarzis (Tunisia) [24]

Material examined: Holotype MNHN HEL11 Th84 [24], MNHN HEL10 Th83 (on the label); voucher specimens newly collected from Tunisia MNHN HEL562. Prevalence: In our specimens from Tunisia, 3/4 (75%).

#### Redescription (Figs 12 and 13; Table 6)

**Fig 12:**
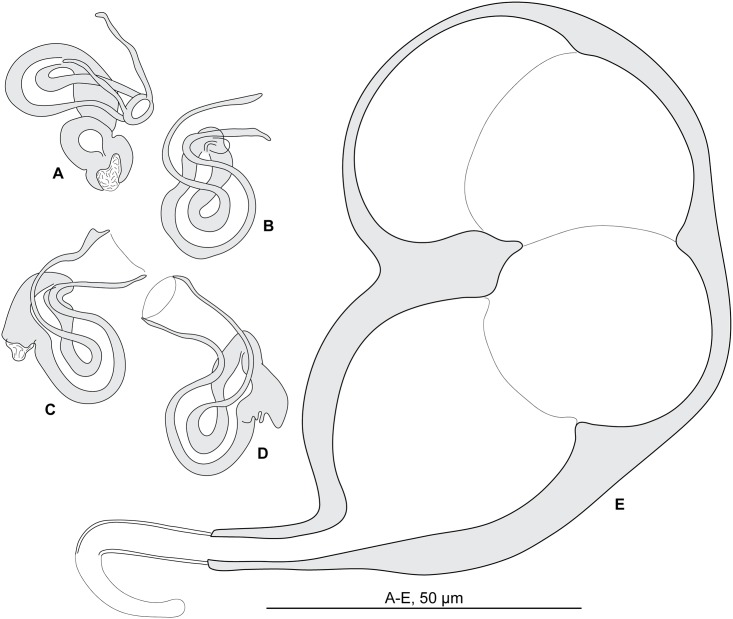
*Pseudorhabdosynochus sinediscus* from *Mycteroperca costae*, quadriloculate organ and various morphologies of vagina. A-D, vaginae; E, quadriloculate organ. All, MNHN HEL562, Tunisia, Berlese.

**Fig 13:**
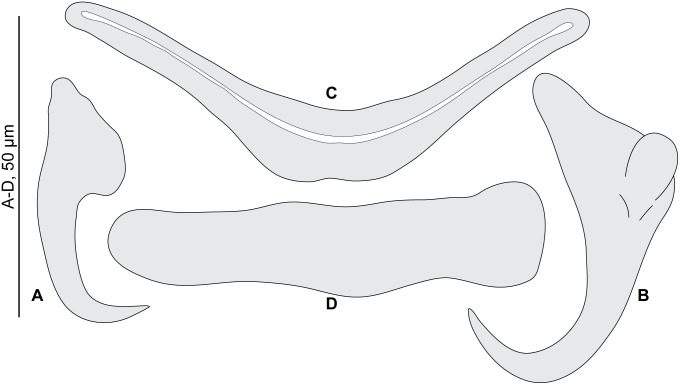
*Pseudorhabdosynochus sinediscus* from *Mycteroperca costae*, haptoral parts. A, dorsal anchor; B, ventral anchor; C, ventral bar; D, lateral bar. All, MNHN HEL562, Tunisia, Berlese. This species has no squamodiscs.

**Table 6:** *Pseudorhabdosynochus sinediscus*, measurements. Means of measurements of sclerotised vaginae, the most important character for systematics, are indicated in bold.

Source	Neifar & Euzet, 2007	Holotype	Vouchers, newly collected specimens
Registration number		MNHN HEL10-Th83	MNHN HEL562
Locality	Off Sfax, off Zarzis, Tunisia (Mediterranean Sea)	Off Sfax, Tunisia (Mediterranean Sea)	Off Sfax, Tunisia (Mediterranean Sea)
Methods	Picrate, Berlese, carmine	Picrate	Berlese
n	13	1	7
Body Length	1,200 (1,050–1,400, n = 10)	-	763 (672–864, n = 3)
Body Width	245 (200–280, n = 10)	-	164 (80–272, n = 4)
Haptor Width	-	-	168 (n = 2)
Pharynx Length	64 (57–70, n = 11)	-	38 (35–40, n = 4)
Pharynx Width	52 (45–60, n = 11)	-	30 (24–35, n = 4)
Penis Internal Length	99 (80–110, n = 10)	75	75 (55–95, n = 7)
Penis Cone Length	24 (20–30, n = 10)	20	19 (16–21, n = 7)
Penis Tube Length	42 (32–55, n = 10)	30	33 (20–45, n = 6)
Penis Tube Diameter		3	4 (4–5, n = 7)
Penis Filament Length		15	17 (15–18, n = 2)
Sclerotized Vagina Total Length	**39** (35–50, n = 18)	**27**	**29** (25–34, n = 7)
Secondary Chamber External Diameter	-	-	-
Ventral Anchor Outer Length	62 (57–68, n = 16)	48 (47–48, n = 2)	46 (42–50, n = 8)
Ventral Anchor Inner Length		47 (46–47, n = 2)	47 (41–50, n = 8)
Dorsal Anchor Outer Length	53 (50–55, n = 16)	42 (41–42, n = 2)	40 (39–42, n = 7)
Dorsal Anchor Inner Length		23 (n = 2)	24 (21–28, n = 7)
Ventral Bar Length	86 (70–95, n = 16)	78	101 (96–104, n = 4)
Ventral Bar Width	13 (10–15, n = 16)	10	14 (12–17, n = 4)
Lateral Bar Length	67 (60–90, n = 16)	52 (51–53, n = 2)	72 (65–78, n = 8)
Lateral Bar Width	14 (10–22, n = 16)	9 (8–9, n = 2)	11 (10–12, n = 8)

**Fig 14:**
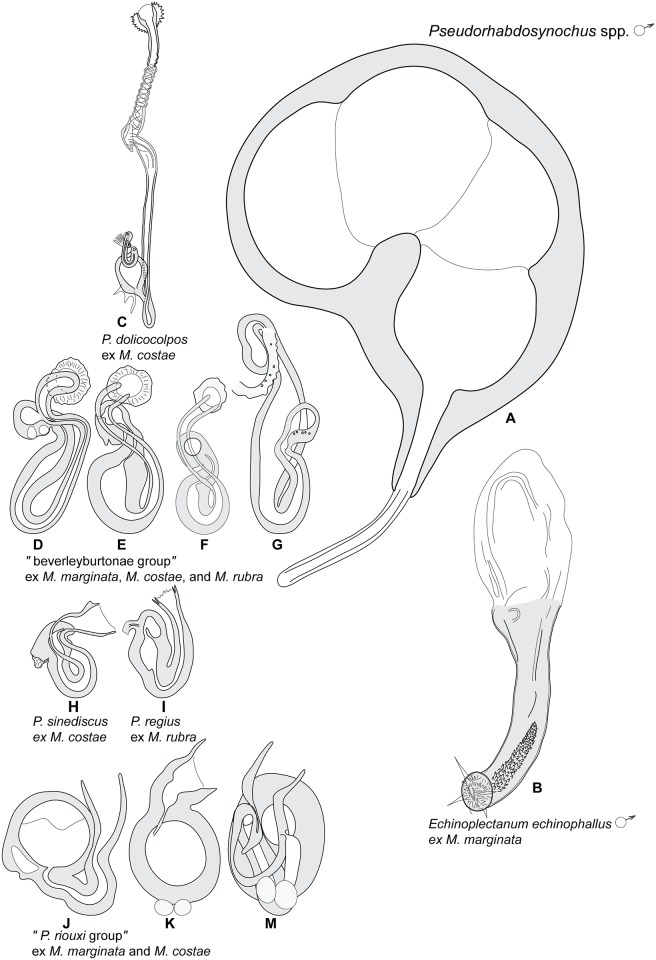
Diplectanid monogeneans on *Mycteroperca* spp. in the Mediterranean Sea. Sclerotized male and female parts. This figure illustrates the taxonomic key (Table 7).

Measurements based on 7 specimens in Berlese and holotype in picrate. Body length b 763 (672–864, n = 3), including haptor; maximum width b 164 (80–272, n = 4) at level of ovary. Tegument smooth. Anterior region with 3 pairs of head organs and 2 pairs of dorsal eye-spots, distance between outer margins of anterior eye-spots b 25 (20–32, n = 6), of posterior eye-spots b 25 (21–31, n = 5). Pharynx median, subspherical, length b 38 (35–40, n = 4), width b 30 (24–35, n = 4). Haptor bearing two pairs of lateral anchors, one ventral bar and two lateral (dorsal) bars (Fig 13) and 14 hooklets, width b 168 (n = 2). Squamodiscs absent. Ventral anchors with distinct handle and guard, outer length h 48, b 46 (42–50, n = 8), inner length h 47, b 47 (41–50, n = 8) (Fig 13A). Dorsal anchors with indistinct guard, outer length h 42, b 40 (39–42, n = 7), inner length h 23, b 24 (21–28, n = 7) (Fig 13B). Lateral (dorsal) bar, with flattened medial end, length h 52, b 72 (65–78, n = 8), maximum width h 9, b 11 (10–12, n = 8) (Fig 13D). Ventral bar, length h 78, b 101 (96–104, n = 4), width h 10, b 14 (12–17, n = 4) (Fig 13C). Male copulatory organ a quadriloculate organ, first (anterior) chamber almost as sclerotized as the three others; fourth chamber forming short cone, prolonged by thin sclerotized tube, inner length b 75 (55–95, n = 7); cone length b 19 (16–21, n = 7); tube length b 33 (20–45, n = 6); tube diameter b 4 (4–5, n = 7), end of tube prolonged by thin unsclerotised filament, length b 17 (15–18, n = 2) (Fig 12E).

*Sclerotized vagina* comprises anterior trumpet, primary canal, primary chamber, and secondary canal (secondary chamber absent). Anterior trumpet, heavily sclerotized, funnel-shaped; primary canal long, S-shaped, its wall slightly thinner distally, opens into posterior part of primary chamber; primary chamber small, heavily sclerotized; very short secondary canal, with thin lumen, emerges from anterior part of primary chamber; secondary chamber not seen, probably very small and embedded in wall of primary chamber (Fig 12A–12D). Total length of sclerotized vagina b 29 (25–34, n = 7).

#### Comments

The inventory number assigned to the holotype of *P*. *sinediscus* as MNHN HEL10-Th83 on the label of the type-slide does not match the one published with the original description of this species (MNHN HEL11-Th84).

#### Differential diagnosis

*Pseudorhabdosynochus sinediscus* is characterized, among its Mediterranean congeners, by the general structure of its vagina in which the secondary chamber is not visible. However, in terms of vaginal morphology, *P*. *sinediscus* is close to *P*. *regius* Chaabane, Neifar & Justine, 2015 from *Mycteroperca rubra* and the ‘beverleyburtonae group’ [22] from *M*. *marginata*, *M*. *costae* and the mottled grouper *M*. *rubra* (Bloch). *Pseudorhabdosynochus sinediscus* and *P*. *regius* are readily distinguished by the shape of their primary canal (S-shaped in *P*. *sinediscus* vs. straight in *P*. *regius*). This species shares the same shape of the vagina with the ‘beverleyburtonae group’ (S-shaped) but differs from it by the small size of its vagina.

In addition, *P*. *sinediscus* is the only species within *Pseudorhabdosynochus* known to have a haptor without squamodiscs. All other currently known species of *Pseudorhabdosynochus* have two squamodiscs. *Pseudorhabdosynochus monosquamodiscusi* Balasuriya & Leong, 1995, from *Lates calcarifer* (Bloch), has a single squamodisc [49], but this species is now considered a member of *Laticola* and a junior synonym of *L*. *latesi* [50].

### *Echinoplectanum echinophallus* (Euzet & Oliver, 1965) Justine & Euzet, 2006

Synonyms: *Diplectanum echinophallus* Euzet & Oliver, 1965; *Cycloplectanum echinophallus* (Euzet & Oliver, 1965) Oliver, 1968.

Type-host: Dusky grouper, *Mycteroperca marginata* (Lowe) (Perciformes, Epinephelidae); synonyms: *Epinephelus guaza* (Linnaeus), *E*. *marginatus* (Lowe).

Molecular identification of fish via DNA barcoding: The COI sequence from one specimen from Tunisia was already published and identified as *M*. *marginata* (Table 1).

Site of infection: Gill lamellae.

Type locality: Off Banyuls, France [51].

Other localities: Mediterranean Sea: Off Lavezzi Island, southern Corsica [52]; off Rosas, Spain [42]; off Sfax, Tunisia ([53] and present paper); eastern Atlantic: Off Dakar, Senegal [16].

Material examined: new specimens collected from Tunisia MNHN HEL560, MNHN HEL591.

Prevalence: In our newly collected specimens from Tunisia, 3/3 (100%).

#### Redescription (Fig 14B)

Measurements based on 6 specimens in Berlese. Body length b 952 (512–1,712, n = 6), width b 164 (128–256, n = 6). Tegument smooth. Anterior region with 3 pairs of head organs and two pairs of eye-spots; distance between outer margins of anterior eye-spot pair b 24 (19–28, n = 6), of posterior eyespot pair b 20 (19–25, n = 6). Haptor bearing two similar squamodiscs, two pairs of lateral anchors, one ventral bar and two lateral (dorsal) bars and 14 hooklets, width b 200 (128–240, n = 6). Squamodiscs round in shape, with numerous concentric rows of rodlets; two innermost rows forming circles. Most rodlets with visible spurs (‘éperons’). Dorsal squamodisc length b 52 (n = 1), width b 65 (n = 1). Ventral anchors with handle and distinct guard, outer length b 60 (59–62, n = 11), inner length b 57 (55–60, n = 11). Dorsal anchors with indistinct guard, outer length b 51 (50–52, n = 8), inner length b 31 (30–35, n = 8). Dorsal (lateral) bars with flattened medial extremity and roughly cylindrical lateral extremity, length b 91 (87–98, n = 12), maximum width b 26 (24–29, n = 12). Ventral bar flat, with constricted median portion, length b 110 (105–115, n = 5), maximum width b 22 (20–24, n = 5). Pharynx not visible. Sclerotized male copulatory organ, funnel-shaped, with muscular reservoir at its anterior extremity with four muscular layers, and spiny cirrus in its posterior part; length b 112 (100–135, n = 5). Sclerotized vagina not seen.

#### Comments

This species was first described by Euzet & Oliver as *Diplectanum echinophallus*, from the dusky grouper in the Mediterranean Sea [51], and later included by Oliver in *Cycloplectanum* Oliver, 1968 due to the presence of two innermost rows forming circles in its squamodiscs [42]. Justine & Euzet (2006) redescribed the species and considered it a member of their new genus *Echinoplectanum* [16]. Among the seven species assigned to *Echinoplectanum* Justine & Euzet, 2006, only *E*. *echinophallus* was not found on the gills of coralgroupers (*Plectropomus* spp.) [16]; all the other species are from the Pacific regions (New Caledonia and Australia).

### Taxonomic key

A taxonomic key to the species of diplectanids on *Mycteroperca* spp. in the Mediterranean Sea is proposed in Table 7. The main morphological features used in the taxonomic key are illustrated in Fig 14.

**Table 7:** Taxonomic key to Diplectanids on *Mycteroperca* spp. in the Mediterranean Sea (Fig 14). Shading of cells applied only for readability.

**1**	• Male copulatory organ with spines. In *Mycteroperca marginata*	*Echinoplectanum echinophallus*	(Fig 14B)
	• Male copulatory organ a quadriloculate organ without spines	*Pseudorhabdosynochus* spp. **2**	(Fig 14A)
**2**	• Sclerotised vagina very elongate, thin, total length >60 μm, with accessory structure on proximal end of secondary chamber. In *Mycteroperca costae*	*Pseudorhabdosynochus dolicocolpos*	(Fig 14C)
	• Sclerotised vagina not elongate, total length <60 μm, without accessory structure	**3**	
**3**	• Secondary chamber visible, heavily sclerotized	**4**	
	• Secondary chamber not visible	**7**	
**4**	• Secondary chamber large and spherical: the “*P*. *riouxi* group”	**5**	(Fig 14J–14M)
	• Secondary chamber small and spherical	**9**	
**5**	• Primary canal very short with wide lumen, opening into proximal limit of primary chamber, primary chamber very small with thick wall. In *Mycteroperca costae*	*Pseudorhabdosynochus bouaini*	(Fig 14K)
	• Primary canal long, opening into distal limit of primary chamber, primary chamber elongate	**6**	
**6**	• Primary canal roughly straight or curved, primary chamber with thick wall. In *Mycteroperca costae*	*Pseudorhabdosynochus enitsuji*	(Fig 14J)
	• Primary canal looped in its proximal or medium portion, primary chamber slightly sclerotized. In *Mycteroperca marginata*	*Pseudorhabdosynochus riouxi*	(Fig 14M)
**7**	• Body without squamodiscs. In *Mycteroperca costae*	*Pseudorhabdosynochus sinediscus*	(Fig 14H)
	• Body with two squamodiscs	**8**	
**8**	• Squamodiscs very small, length 20–40 μm, with innermost rows U-shaped. In *Mycteroperca rubra*	*Pseudorhabdosynochus regius*	(Fig 14I)
	• Squamodiscs length >40 μm, with 2 innermost rows forming circles. The ‘beverleyburtonae group’ (Fig 14D–14G)	**9**	
**9**	• Primary canal, S-shaped with wide lumen, leading to proximal limit of primary chamber. In *Mycteroperca costae*	*Pseudorhabdosynochus oliveri*	(Fig 14G)
	• Primary canal, S-shaped with thin lumen, leading to distal limit of primary chamber	**10**	
**10**	• Primary chamber elongate. In *Mycteroperca marginata*	*Pseudorhabdosynochus beverleyburtonae*	(Fig 14D)
	• Primary chamber pear-shaped	**11**	
**11**	• Length of sclerotised vagina 26–58 μm. In *Mycteroperca costae*	*Pseudorhabdosynochus sosia*	(Fig 14E)
	• Length of sclerotised vagina 40–70 μm. In *Mycteroperca rubra*	*Pseudorhabdosynochus hayet*	(Fig 14F)
